# Large enhancement of superconducting transition temperature of SrBi3 induced by Na substitution for Sr

**DOI:** 10.1038/srep10089

**Published:** 2015-05-12

**Authors:** Akira Iyo, Yousuke Yanagi, Tatsuya Kinjo, Taichiro Nishio, Izumi Hase, Takashi Yanagisawa, Shigeyuki Ishida, Hijiri Kito, Nao Takeshita, Kunihiko Oka, Yoshiyuki Yoshida, Hiroshi Eisaki

**Affiliations:** 1 National Institute of Advanced Industrial Science and Technology (AIST), 1-1-1 Umezono, Tsukuba, Ibaraki 305-8568, Japan; 2 IMRA Material R&D Co., Ltd., 2-1 Asahi-machi, Kariya, Aichi 448-0032, Japan; 3 Department of Physics, Tokyo University of Science, 1-3 Kagurazaka, Shinjuku, Tokyo 162-8601, Japan

## Abstract

The Matthias rule, which is an empirical correlation between the superconducting transition temperature (*T*_c_) and the average number of valence electrons per atom (*n*) in alloys and intermetallic compounds, has been used in the past as a guiding principle to search for new superconductors with higher *T*_c_. The intermetallic compound SrBi_3_ (AuCu_3_ structure) exhibits a *T*_c_ of 5.6 K. An *ab-initio* electronic band structure calculation for SrBi_3_ predicted that *T*_c_ increases on decreasing the Fermi energy, i.e., on decreasing *n*, because of a steep increase in the density of states. In this study, we demonstrated that high-pressure (~ 3 GPa) and low-temperature ( < 350 °C) synthesis conditions enables the substitution of Na for about 40 at.% of Sr. With a consequent decrease in *n*, the *T*_c_ of (Sr,Na)Bi_3_ increases to 9.0 K. A new high-*T*_c_ peak is observed in the oscillatory dependence of *T*_c_ on *n* in compounds with the AuCu_3_ structure. We have shown that the oscillatory dependence of *T*_c_ is in good agreement with the band structure calculation. Our experiments reaffirm the importance of controlling the number of electrons in intermetallic compounds.

In various transition metals, their alloys, and intermetallic compounds, an empirical correlation exists between *T*_c_ and the average number of valence electrons (electrons outside of closed shells) per atom, *n*. The *n* is simply calculated from a chemical formula. For example, the total valence electrons in Nb_3_Ge are 19 because Nb and Ge atoms have 5 and 4 electrons outside of closed shells, respectively. Therefore, the *n* is calculated to be 4.75 by dividing 19 by 4 (the number of atoms in the formula). The most notable examples are 4*d* transition metal alloys and the A15 (Cr_3_Si structure) compounds^1^,^2^,^3^, among which materials with *n* = 4.7 or 6.7 tend to exhibit high *T*_c_. For Nb_3_Ge, the material with the highest *T*_c_ (=23.9 K) among the A15 compounds^4^, *n* = 4.75. It is argued that this empirical rule, called the Matthias rule, is associated with the characteristic shape of the density of states *N*(*E*), which exhibits sharp peaks corresponding to these electron numbers^5^,^6^.

In principle, the above empirical rule should hold for various types of superconductors. Therefore, the *T*_c_ of superconductors with given crystal/electronic structures can theoretically be increased by tuning the electron number *n* so as to maximize *N*(*E*) at the Fermi energy *E* _F_. In this study, we demonstrated that this guiding principle holds for a real material, SrBi_3_ (*T*_c_ = 5.62 K^7^). This intermetallic compound is crystallized into an AuCu_3_ structure as shown in Fig. 1. According to an *ab-initio* electronic band structure calculation, the *N*(*E*) around *E*_F_ of SrBi_3_ is dominated by the Bi 6*p* orbitals (Fig. 1). A sharp peak in *N*(*E*) is located 0.02 Ry below *E*_F_ , as indicated by an arrow in Fig. 1. Assuming a rigid band model, one expects that *T*_c_ would be increased on decreasing *E*_F _, which is realized by decreasing *n*.

The most straightforward way to decrease *n* in SrBi_3_ is to replace divalent Sr ions with monovalent alkali metal ions, such as K or Na. However, to our knowledge, such substitution has not successfully been performed in usual intermetallic compounds, because alkali metals are far more reactive compared to alkaline earth metals. The synthesis conditions of the substituted samples are thus entirely different from those of the pristine ones; in particular, the substituted samples require low-temperature and tightly sealed conditions in order to prevent the evaporation of volatile alkali metals. We realized such conditions by using a cubic-anvil-type high-pressure (HP) apparatus and succeeded in synthesizing (Sr,Na)Bi_3_. As expected, the *T*_c_ of (Sr,Na)Bi_3_ increases with Na concentration, reaching up to 9.0 K. Based on the present results, we demonstrated that there is a new and higher peak in the oscillatory relationship between *T*_c_ and *n* for materials with an AuCu_3_ structure and that the relationship results from the *n*-dependence of *N*(*E*), which is characteristic of this crystal structure.

## Results

### Introduction of Na and enhancement in *T*_c_ through the high-pressure, low-temperature synthesis

Figure 2 shows the χ(*T*) of the samples with nominal compositions of (Sr_1-*x*_Na_1.5*x*_)Bi_3_ (*x* = 0.5) synthesized under various temperatures (*T*_syn_) ranging from 300–450 °C for 6 h. For the case of *T*_syn_ = 350 and 300 °C, samples were slowly cooled (20 °C/h) to 225 and 200 °C, respectively. All the samples exhibit higher *T*_c_ compared to pristine SrBi_3_ (*T*_c_ = 5.6 K). Moreover, *T*_c_ increases on lowering *T*_syn_, up to 9.0 K (indicated by an arrow) for *T*_syn_ = 300 °C. The *T*_c_ of 9.0 K is the second highest among the superconductors possessing the AuCu_3_ structure, the highest being *T*_*c*_ = 9.54 K for InLa_3_^8^.

The lattice parameter of the samples, *a*, decreases with decreasing *T*_syn_; for example, *a* = 5.013, 4.992, and 4.989 Å for *T*_syn_ = 450, 400 and 300 °C, respectively. (*a* = 5.04 Å for SrBi_3_). The decrease in the lattice parameter is due to the increase in Na substitution at the Sr sites, as will be elaborated in a later section.

It should be noted that HP, low-temperature synthesis conditions promote the Na substitution for Sr. Indeed, the sample synthesized under ambient conditions (*x* = 0.5, annealed at 300 °C in an evacuated quartz tube) exhibits a lower *T*_c_ of 6.5 K and larger *a* of 5.033 Å.

### Change in the structure upon Na substitution: powder X-ray diffraction patterns

As the next step, we synthesized series of samples with various values of *x* – 0, 0.2, 0.4, 0.5, and 0.6 – by reacting the starting materials at 350 °C for 6 h, following which annealing was performed at (or with slow cooling down to) 275–225 °C for 6 h at a pressure of 3.4 GPa.

The powder XRD patterns of the reacted samples are shown in Fig. 3. Major peaks can be indexed on the basis of the cubic unit cell expected for the AuCu_3_ structure. The *a* value of SrBi_3_ (*x* = 0) calculated using a least-squares fitting is 5.043 Å, which is in good agreement with the previously reported value (5.035–5.04 Å)^9^. The overall X-ray patterns do not change with increase in *x*, while the peak width broadens, which is due to the disorder associated with the Na substitution at the Sr sites. Diffraction peaks corresponding to Bi are observed for all samples, with their intensity increasing with *x*. For the samples with *x* = 0.5 and 0.6, peaks corresponding to NaBi are also observed, indicating that the introduced Na is not completely incorporated into the samples and that the solubility limit is around *x* = 0.4–0.5.

### The *x*-dependence of lattice parameter *a* and *T*_c_

Figure 4(a) shows χ(*T*) for the samples with various values of *x*. SrBi_3_ (*x* = 0) shows a sharp superconducting transition at 5.6 K, which is in good agreement with the reported value. As *x* increases, *T*_c_ monotonously shifts to higher temperatures. Note that the transition width does not change with changing *x*, suggesting that Na is uniformly incorporated into the samples. The shielding volume fraction calculated from the ZFC susceptibility value at 5 K exceeded 100% for all samples, indicating bulk superconductivity.

In Fig. 4(b), the lattice parameter *a* and *T*_c_ are plotted as functions of *x*. The lattice parameter *a* decreases linearly with *x* up to *x* *=* 0.3 and then saturates above *x* *=* 0.4. The decrease of *a* results from the substitution of smaller Na^+^ (with ionic radius (XII coordination) of 1.39 Å^10^) into the Sr^2+^ (1.44 Å) sites. The substitution of larger K^+^ (ionic radius of 1.64 Å) into the Sr^2+^ sites was not successful. As seen in Fig. 4(b), *T*_c_ and *a* exhibit similar *x*-dependences; they change linearly with *x* up to *x* = 0.3 and then saturate in the vicinity of *x* = 0.4. These behaviours again indicate that the Na solubility limit is around *x* = 0.4.

## Discussion

In the present study, we demonstrated that the *T*_c_ of SrBi_3_ increases with decreasing *n*. The relationship between *T*_c_ and *n* for compounds with the AuCu_3_ structure was first discussed by Havinga *et al.*^9^. They showed that *T*_c_ exhibits an oscillatory dependence on *n* with peaks at *n* = 3.75 and 4.00. For example, LaSn_3_ (*T*_c_ = 6.02 K) and ThPb_3_ (*T*_c_ = 5.55 K) have *n* = 3.75 and 4.00, respectively. Most known superconductors with the AuCu_3_ structure have *n* values in the range 2.75 ≤ *n* ≤ 4.00. SrBi_3_ (*n* = 4.25) has an exceptionally large *n*, which enabled us to investigate *T*_c_ for 4.00 ≤ *n* ≤ 4.25.

**Figure 1:**
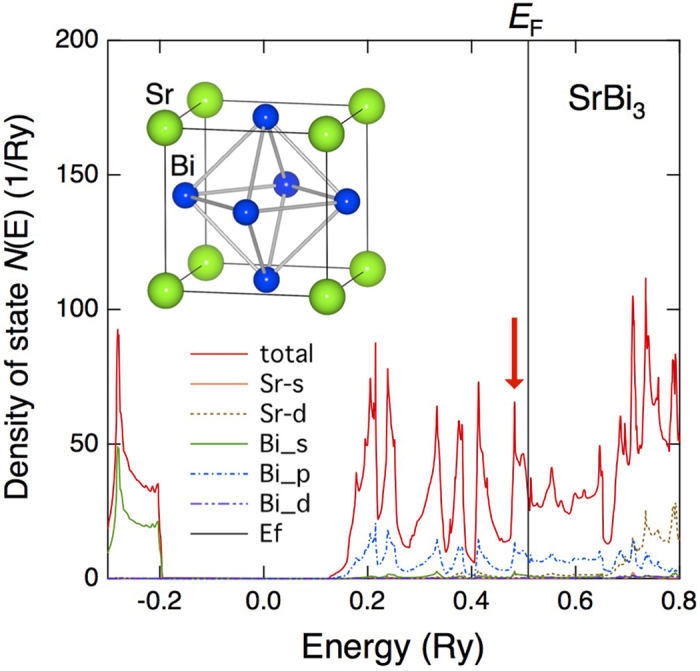
Density of states (*N*(*E*)) of SrBi_3_ obtained through an *ab-initio* electronic band structure calculation. An illustration of the crystal structure of SrBi_3_ (AuCu_3_ structure) is shown in the figure. Sr and Bi atoms occupy the cube corners and face centres, respectively.

**Figure 2:**
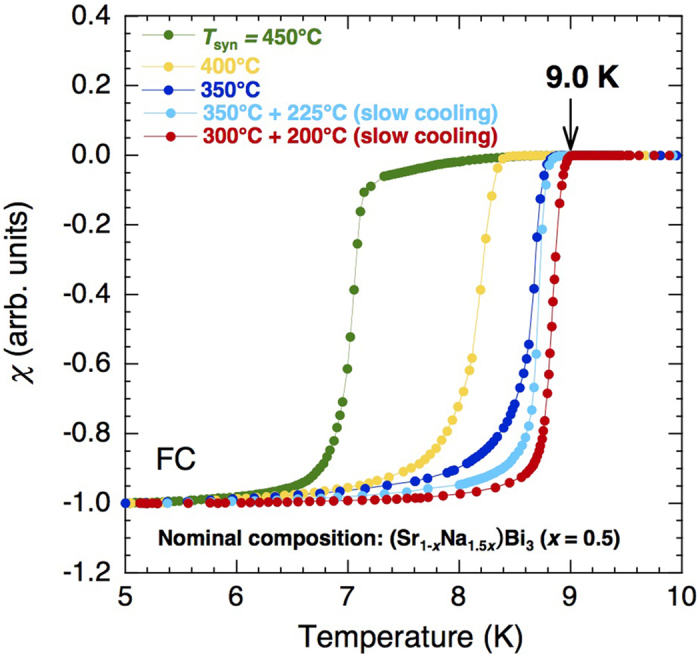
Temperature (T) dependence of normalized field-cooled (FC) susceptibilities χ(T) for samples with nominal compositions of (S_r1-x_Na_1.5x_)Bi_3_ (x = 0.5) synthesized at various temperatures (T_syn_). χ(T) is normalized between 5 and 10 K for clarity. As Tsyn decreases, Tc increases, reaching up to 9.0 K, as indicated by an arrow.

**Figure 3:**
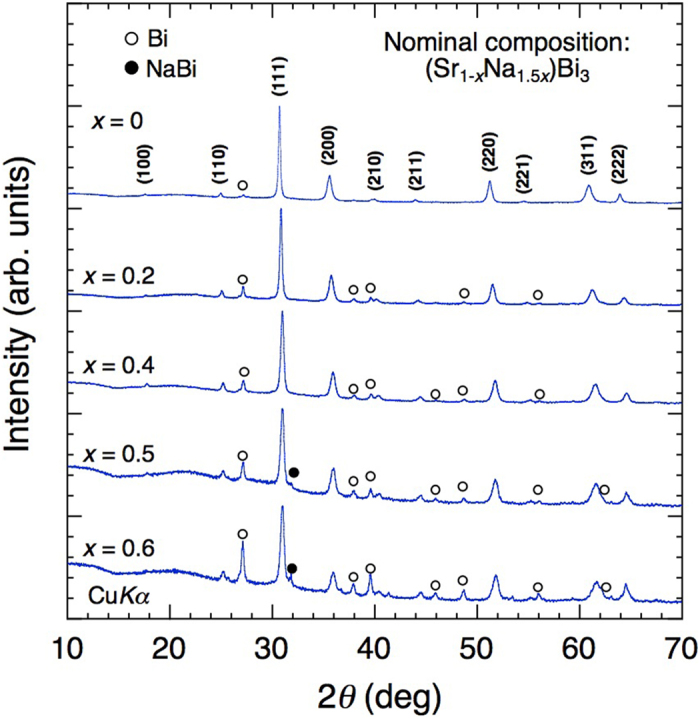
Powder X-ray diffraction patterns of the samples with nominal compositions of (S_r1-x_Na_1.5x_)Bi_3_ (x = 0, 0.2, 0.4, 0.5, and 0.6). Peaks are indexed as the cubic unit cell of the AuCu_3_ structure. The diffraction peaks indicated by open and closed circles correspond to Bi and NaBi, respectively.

**Figure 4:**
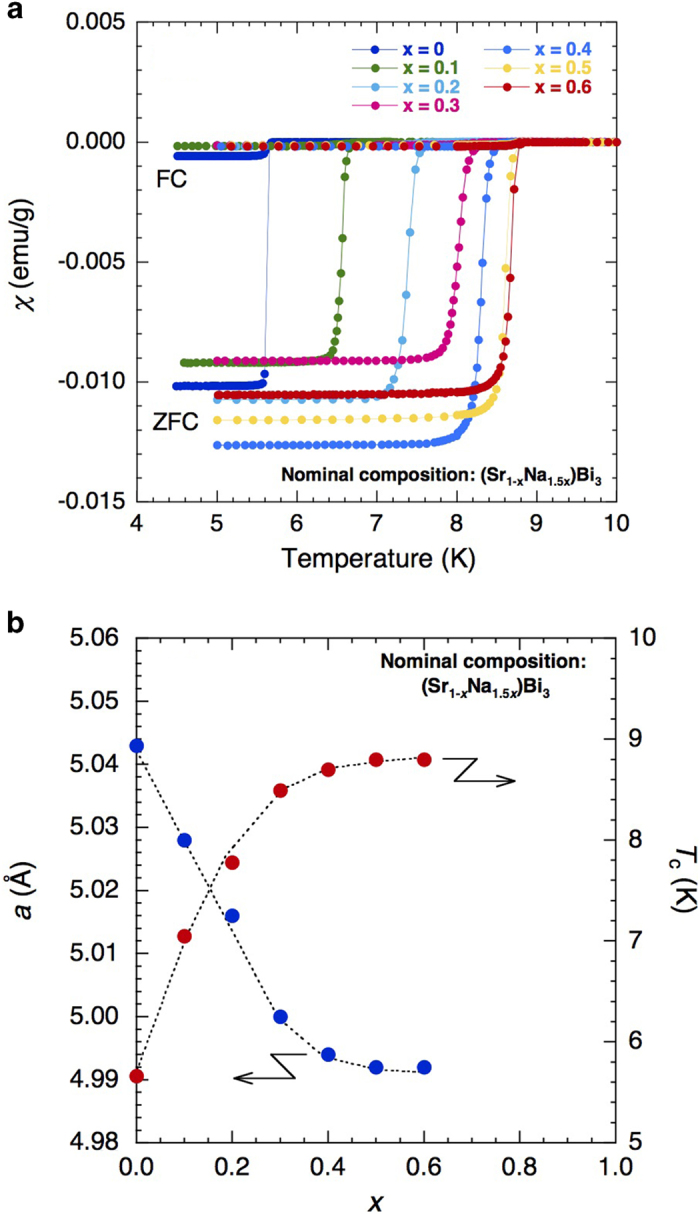
(**a**) T-dependence of magnetic susceptibility χ(T) for the samples with nominal compositions of (S_r1-x_Na_1.5x_)Bi_3_ (x = 0, 0.1, 0.2, 0.3, 0.4, 0.5, and 0.6). (**b**) Plot of the lattice parameter *a* and *T*_c_ as functions of x. The dashed curves are guides for the eye.

**Figure 5:**
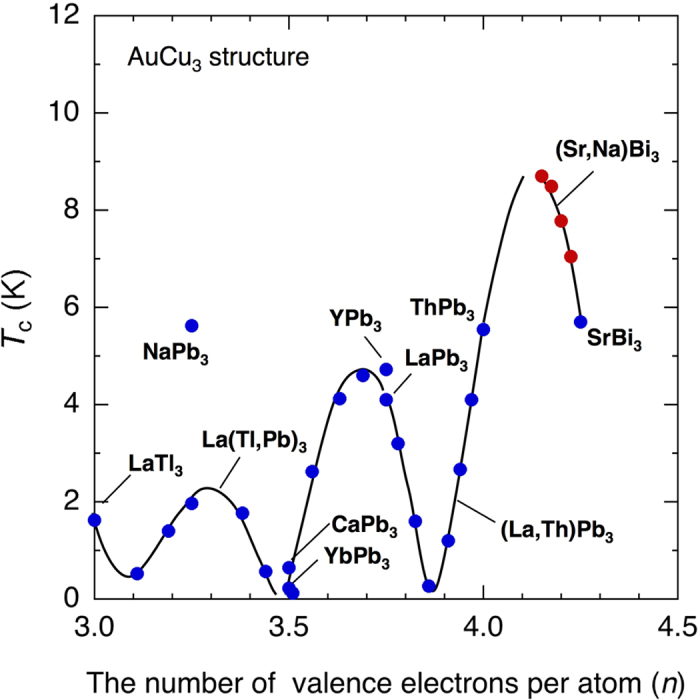
Relationship between *n* and T_c_ of superconductors with the AuCu_3_ structure that contain Pb, Bi, or Tl at the Cu site. Data of the present study are added to those taken from Havinga *et al*^9^.

Figure 5 shows the general relationship between *n* and *T*_c_ for superconductors with the AuCu_3_ structure that contain Pb, Bi, or Tl at the crystallographic Cu site. Their band structures near *E*_F_ are similar; the band structures are dominated by Bi (or Pb, Tl) 6*p* orbitals, as shown in Fig. 1. In Fig. 5, one can recognize a clear oscillation of *T*_c_ with respect to *n*. In particular, a new *T*_c_ peak corresponding to (Sr,Na)Bi_3_ is observed at *n* ~ 4.15. This peak is higher than those located around *n* = 3.25 and 3.70. These *T*_*c*_ peaks reflect the shape of *N*(*E*) shown in Fig. 1. In Fig. 1, the *N*(*E*) peak immediately below *E*_F_ corresponds to *n* = 4.0, and the next two *N*(*E*) peaks at energies of ~ 0.41 Ry and ~ 0.38 Ry correspond to *n* ~ 3.5 and *n* ~ 3.25, respectively. These peaks are mainly attributed to the Bi-6*p*π-bonding bands, which have narrow band widths. Considering the crudeness of the rigid band model, the agreement between the experiments and theory is reasonably good.

In summary, we have demonstrated that the *T*_c_ of SrBi_3_ increases by tuning the number of valence electrons on the basis of the prediction from the band structure calculation. High-pressure and low-temperature synthesis conditions enables to substitute a large amount of Na for Sr in SrBi_3_. Consequently, the *T*_c_ of SrBi_3_ increases from 5.6 K to as high as 9.0 K by decreasing the *n*. We have shown that a new high oscillation peak appears on the *n* dependence of *T*_c_ in compounds with the AuCu_3_ structure and the *T*_c_ oscillation is in good agreement with the band structure.

## Methods

### Electronic band structure calculation

Our calculation is based on the local-density approximation (LDA) and implemented using the computer code KANSAI-94 and TSPACE^11^. Spin-orbit interaction is included in a second-variational procedure. We used the experimental value of 5.043 Å for the lattice parameter *a*. The muffin-tin radii were set as 0.252a for Sr and 0.231a for Bi.

### Preparation of (Sr,Na)Bi_3_ samples

Series of polycrystalline samples of (Sr,Na)Bi_3_ were synthesized through a solid-state reaction by using a cubic-anvil-type HP apparatus. The starting materials were Bi, Na_3_Bi, and Sr_5_Bi_3_ powders. Na_3_Bi powder was prepared by heating an appropriate amount of Na and Bi chunks at 900 °C in an alumina crucible sealed in a stainless-steel vessel^12^. Sr_5_Bi_3_ was prepared by heating a mixture of Bi and Sr powders to 950 °C by using the HP synthesis method or a method similar to that used for preparing Na_3_Bi powder.

An appropriate amount of the starting materials were ground with agate mortar in a nitrogen-filled glove box and pressed into a pellet. Excess Na (50%) was added to the starting compositions to compensate for possible Na loss during the heat treatment. The pellet with a nominal composition of (Sr_1-x_Na_1.5x_)Bi_3_ was placed in a BN crucible and assembled into an HP cell^13^. The sample was heated under a pressure of 3.4 GPa. As we have described, the *T*_c_ of samples strongly depends on the sample synthesis temperature (*T*_syn_); *T*_*c*_ monotonously increases with decreasing *T*_syn_, even when starting from the same nominal values of *x*. The resulting samples were handled in a nitrogen- or argon-filled glove box because of their reactivity in air.

### Material characterization

Powder X-ray diffraction (XRD) patterns were measured at room temperature using Cu *K*_α_ radiation. Because the samples are easily degraded by reactions with oxygen and/or moisture in air, a polyimide adhesive tape was placed on the sample, and the XRD pattern was collected for 8 min using a diffractometer equipped with a high-speed detector system (Rigaku, D/teX Ultra). Temperature (*T*)-dependent magnetic susceptibility (χ(*T*)) measurement was performed using a magnetic property measurement system (MPMS) (Quantum Design, MPMS-XL7) under a magnetic field of 0.001 T. The data were collected during warming after zero-field cooling (ZFC) and then during field cooling (FC). *T*-dependent electrical resistivity (ρ(*T*)) was measured using the four-probe method under magnetic fields of up to 2.4 T. Because the sample is unstable in air, the electrodes were coated with a silver paste in an argon-filled glove box and covered with APIEZON grease before exposure to air.

## Author Contributions

A. I., Y. Y., T. K., S. I., H. K., and K. O. performed the sample preparation and characterization. A. I., T. N., Y. Y., and H. E. drafted the manuscript. T. K., T. N., and N. T measured superconducting properties of samples. I. H. and T. Y. performed an *ab-initio* electronic band structure calculation. All authors reviewed and approved the manuscript.

## Additional Information

**How to cite this article**: Iyo, A. *et al.* Large enhancement of superconducting transition temperature of SrBi3 induced by Na substitution for Sr. *Sci. Rep.* **5**, 10089; doi: 10.1038/srep10089 (2015).
